# Lung perfusion findings on perfusion SPECT/CT imaging in non-hospitalized de-isolated patients diagnosed with mild COVID-19 infection

**DOI:** 10.1186/s43055-021-00521-1

**Published:** 2021-06-09

**Authors:** Osayande Evbuomwan, Gerrit Engelbrecht, Melissa V. Bergman, Sello Mokwena, Oluwatosin A. Ayeni

**Affiliations:** 1 Nuclear Medicine Department, University of The Free State, Universitas Academic Hospital, Lower ground floor, Logeman Street, Bloemfontein, 9301 South Africa; 2 Universitas Academic Hospital, Bloemfontein, South Africa; 3 Non-Communicable Diseases Research Division, Wits Health Consortium (Pty) Ltd, University of The Witwatersrand, Johannesburg, South Africa

**Keywords:** VQ SPECT/CT, Perfusion defects, COVID-19, Perfusion abnormalities, Mosaic attenuation

## Abstract

**Background:**

The aim of this retrospective study is to assess the incidence and type of lung perfusion abnormalities in non-hospitalized patients diagnosed with mild COVID-19 infection after de-isolation. Data from 56 non-hospitalized patients diagnosed with COVID-19 infection referred to our nuclear medicine department from July–December 2020 for a perfusion only SPECT/CT study or a ventilation perfusion SPECT/CT study were collected. Images were assessed for the presence and type of perfusion defects. The CT component of the study was also assessed for the presence of mosaic attenuation and COVID pneumonia changes.

**Results:**

Thirty-two (57.1%) cases had perfusion defects. There were 20 (35.7%) cases with defects in keeping with pulmonary embolism, 17 (30.4%) cases with defects associated with mosaic attenuation but not due to pulmonary embolism, and 6 (10.7%) of cases with defects due to pulmonary infiltrates from COVID pneumonia. A total of 24 (42.9%) cases had mosaic attenuation on CT, with 10 (17.9%) of them showing a pattern likely consistent with shunting on the perfusion images.

**Conclusion:**

Lung perfusion abnormalities are a common finding in non-hospitalized COVID-19 patients with mild disease. They are usually either due to pulmonary embolism, parenchymal infiltrates, or other causes of mosaic attenuation related to, but not specific to the pathophysiology of COVID-19 infection. The value of VQ SPECT/CT imaging is also shown in this study, in detecting and differentiating the various types of perfusion abnormalities.

## Background

Severe acute respiratory syndrome coronavirus 2 (SARS-CoV-2) is a novel coronavirus responsible for the ongoing coronavirus disease 2019 (COVID-19) global pandemic. This infection results in a wide spectrum of clinical manifestations including flu-like symptoms, difficulty in breathing, blood and circulatory complications, gastrointestinal symptoms, and others [1]. The pathophysiology of SARS-CoV-2 is becoming clearer now, with its major symptoms being severe acute respiratory syndrome due to virus interaction with cells expressing angiotensin-converting enzyme 2 (ACE2) in the lungs [2]. This interaction is thought to involve ACE2-mediated cellular viral entry, tissue damage, dysregulation of the renin-angiotensin-aldosterone system, systemic release of cytokines, and dysfunctions in the microcirculation [3, 4]. In the lungs, there might be further accumulation of immune cells with overproduction of pro-inflammatory cytokines and activation of complement and coagulation systems in lung microcirculation [2].

Some studies have shown that processes other than alveolar damage might be responsible for hypoxemia seen in patients with COVID-19 infection, an example being the presence of microvascular thrombi [5]. This has been shown to be consistent with elevated D-dimer levels and the presence or absence of microvascular disease like pulmonary embolism (PE) [6]. Studies have also shown associated lung perfusion abnormalities in patients with severe COVID-19 infection presenting with various respiratory symptoms [2, 5–8]. These perfusion abnormalities may include mosaic perfusion, focal hyperemia in a subset of pulmonary opacities, and focal oligemia associated with a subset of peripheral opacities [7].

A ventilation perfusion (VQ) scan is a well-known and commonly performed study in most nuclear medicine facilities. This study is commonly used in identifying perfusion defects in keeping with PE [9–13]. Due to the potential spread of infection to nuclear medicine health workers during the ventilation of patients, some nuclear medicine centers abolished the ventilation part of the study [10]. Our facility initially took this precaution in the earlier months of the pandemic.

Anecdotal evidence in our facility had shown persistent respiratory symptoms in some non-hospitalized patients with mild COVID-19 infection after de-isolation. This study aims to assess the type and incidence of lung perfusion defects that might be responsible for persistent respiratory symptoms in non-hospitalized patients with mild COVID-19 infection after de isolation with VQ and/or perfusion only single photon emission computed tomography/computed tomography (SPECT/CT) imaging.

## Methods

Ethics approval was obtained from the appropriate ethics committee.

### Study design

The study was a retrospective cohort study.

### Study population

Data of fifty-six non-hospitalized patients diagnosed with mild COVID-19 infection after de-isolation were analyzed. All patients had raised D-dimer levels and persistent or new onset respiratory symptoms after de-isolation. None of these patients had prior hospitalization for severe disease. Forty-seven patients had a perfusion only SPECT/CT study as their initial study, while nine patients had a VQ SPECT/CT study as their initial study. This was because earlier on in the pandemic, we were cautious with ventilating patients, because of the potential spread of infection. Fourteen patients had a follow-up VQ SPECT/CT study 3 months after initiation of therapeutic anticoagulation for PE. The recruitment period for the study was between August 2020 and January 2021.

### Equipment

Ventilation studies were performed with 20–25mCi of technetium 99 metastable diethylenetriamine pentacetate (^99m^Tc-DTPA) using the SmartVentTM (Diagnostic Imaging Limited UK) radioaerosol delivery system. Perfusion study was performed with 3–5mCi of ^99m^Tc macro-aggregated albumin (MAA).

Images were acquired with either a 16-slice SPECT/CT camera (Siemens Symbia T16 True point SPECT-CT; Siemens medical solutions, USA) or a 2-slice SPECT/CT camera ((Siemens Symbia T True point SPECT-CT; Siemens medical solutions, USA). Both cameras are dual headed gamma cameras, with similar work stations and processing units.

### Acquisition protocol

Both gamma cameras were equipped with a low energy high resolution (LEHR) collimator. For those patients who had ventilation studies performed, SPECT imaging was acquired immediately after ventilation of the radioaerosol at 15s/stop, with 3° steps, in a 128×128 matrix. Then perfusion SPECT imaging was acquired after injection of the perfusion tracer at 12s/stop, with 3° steps, in a 128×128 matrix. This was followed by low dose non contrast chest CT, with the patient remaining in the same position.

### Image processing

Images were processed using the Syngo work stations for both gamma cameras. SPECT images were reconstructed using an iterative algorithm and SPECT/CT fusion images were obtained using the multimodality Syngo imaging software on the work station.

### Data analysis

Data from each patient was collected using Microsoft Excel 2019 and was analyzed using the statistical package Stata version 16 (StataCorp Ltd, TX, USA).

### Statistical analysis

The results of the following variables—sex, study type, cases with follow-up studies, presence of defects, cases with PE, cases with mosaic attenuation, cases with shunting, and cases with COVID pneumonia—were represented as frequencies and percentages. Age was represented as the median and interquartile range (IQR).

Binary logistic regression was used to determine the association of age with the three types of defects (PE, mosaic attenuation, pulmonary infiltrate), reporting odds ratios (OR), and 95% confidence intervals (CI). A two-sided p-value of <0.05 was considered significant throughout.

## Results

**Fig. 1:**
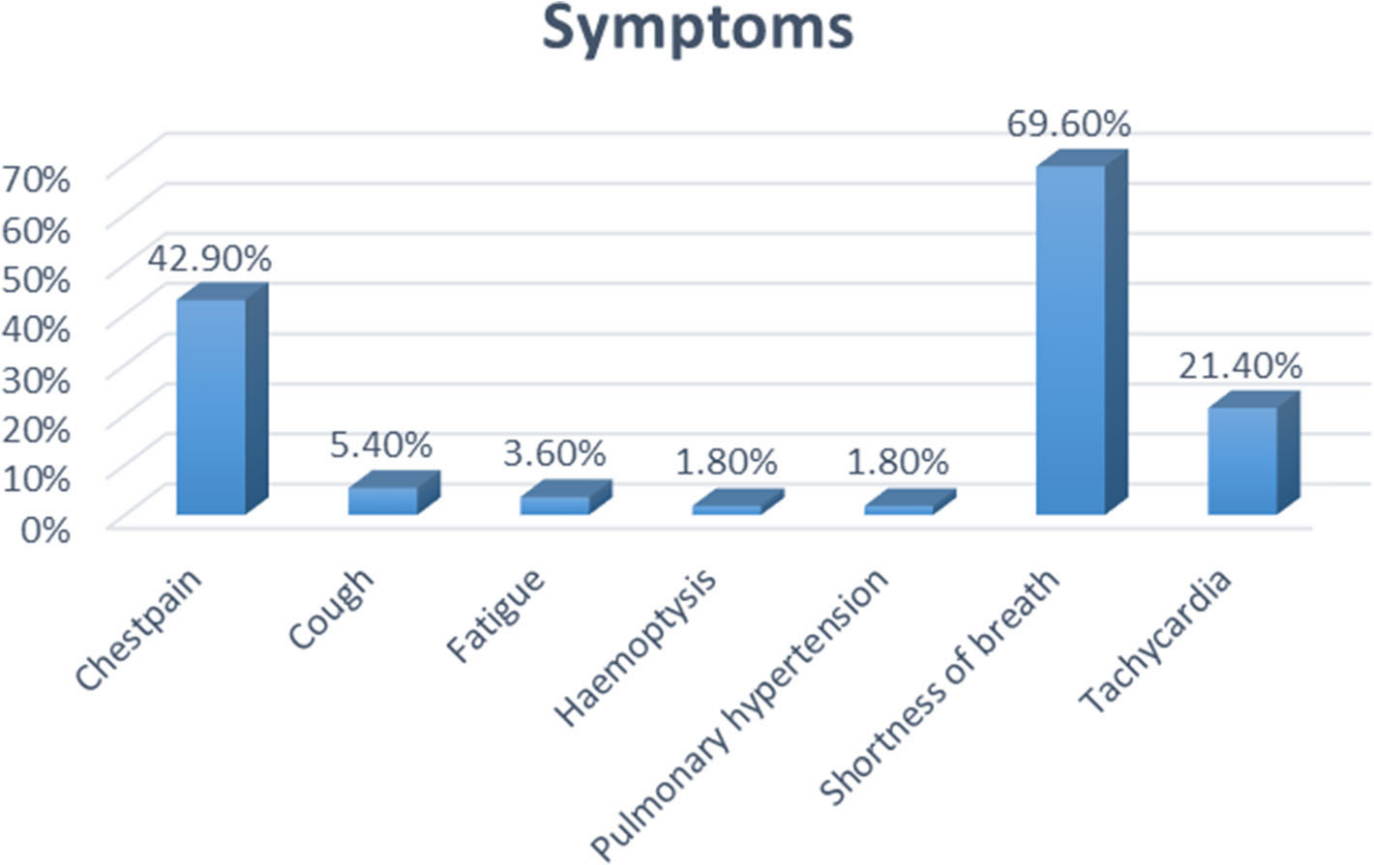
Distribution of symptoms prompting referral for the study

Fifty-six patients were enrolled during the study period. The median (IQR) age was 48 (41–54) years and the majority (87.5%) were females (Table 1). All participants had raised D-dimer levels with new onset or persistence of respiratory symptoms. The most common symptom was shortness of breath (69.6%), followed by chest pain (42.9%) and tachycardia (21.4%) (Fig. 1).

**Table 1:** Demographic and clinical characteristics

Characteristics	N=56
**Age in years,** ***median (IQR)*** ^***a***^	48 (41–54)
**Sex,** ***n (%)***	
Female	49 (87.5)
Male	7 (12.5)
^**a**^ **Duration of illness before scan in days,** ***median (IQR)***	17 (14–25)
^**b**^ **Study type,** ***n (%)***	
Q SPECT/CT scan	47 (83.9)
VQ SPECT/CT scan	9 (16.1)
^**b**^ **Follow-up study,** ***n (%)***	
None	42 (75.0)
VQ SPECT/CT scan	14 (25.0)
**Defects,** ***n (%)***	
No	24 (42.9)
Yes	32 (57.1)
**Mosaic attenuation,** ***n (%)***	
No	32 (57.1)
Yes	24 (42.9)
**COVID pneumonia*, n (%)***	
No	32 (57.1)
Yes	24 (42.9)
^**b**^ **Defects from PE,** ***n (%)***	
No	31 (55.4)
Yes	20 (35.7)
Non-diagnostic	5 (8.9)
^**b**^ **Defects with mosaic attenuation other than PE,** ***n (%)***	
No	39 (69.6)
Yes	17 (30.4)
**Defects from pulmonary infiltrates,** ***n (%)***	
No	50 (89.3)
Yes	6 (10.7)
**Shunting,** ***n (%)***	
No	46 (82.1)
Yes	10 (17.9)

^***a***^IQR (interquartile range)

^b^*Q SPECT/CT* perfusion single photon emission computed tomography/computed tomography, *VQ SPECT/CT* ventilation and perfusion single photon emission computed tomography/computed tomography, *PE* pulmonary embolism

All the patients had lung perfusion studies performed on de-isolation, 10–90 days after the diagnosis of COVID-19 infection, with 47 (83.9%) of them having perfusion only SPECT/CT study, while only 9 (16.1%) had a VQ SPECT/CT study. Of these 56 participants, 14 (25.0%) of them had a follow-up VQ SPECT/CT study performed 3 months after their initial study. Table 1 shows the demographics and proportion of cases with lung perfusion defects, type of lung perfusion defects, and presence of COVID pneumonia. There were 2 false-positive cases of PE diagnosed by an initial perfusion only SPECT/CT study. Four cases had defects wrongly diagnosed as due to PE on initial perfusion only SPECT/CT. However, this did not change the diagnosis of PE in two of these cases as they also had other associated true defects that were in keeping with PE.

The median (IQR) age in years of those with perfusion defects was not significantly different from those without perfusion defects (50 (43–60) years vs. 44 (39–54), p=0.090). Patients with defects not due to PE but associated with mosaic attenuation were significantly older than those without these defects, median (IQR) age in years (53 (43–60) vs. 44 (39–53)). For every 1-year increase in age, there was an 8% increased odds ratio of presenting with defects associated with mosaic attenuation (OR 1.08, 95% CI 1.01–1.15, p=0.023). The older the patients were, the less likely they were to present with defects from pulmonary infiltrates, though this was not significant (OR 0.93, 95% CI 0.84–1.03, p=0.147) (Table 2).

**Table 2:** Association of age with all three types of perfusion defect

Study findings	No	Yes	OR (95% CI)	P value
	**Defects from pulmonary embolism**			
	N=36	N=20		
**Age in years, median (IQR)**	44 (39–54)	50 (43–60)	1.05 (0.99–1.12)	0.090
	**Defects from mosaic attenuation**			
	N=39	N=17		
**Age in years, median (IQR)**	44 (39–53)	53 (43–60))	1.08 (1.01–1.15)	**0.023**
	**Defects from pulmonary infiltrate**			
	N=50	N=6		
**Age in years, median (IQR)**	47 (41–57)	43 (34–45)	0.93 (0.84–1.03)	0.147

## Discussion

Pulmonary perfusion and vessels are frequently abnormal in COVID-19 pneumonia and may point to a key role of pulmonary vascular pathology and hypoxemia in COVID-19 infection [7]. Studies have shown associated lung perfusion abnormalities in patients with severe COVID-19 infection presenting with various respiratory symptoms [2, 5–8]. These perfusion abnormalities may include mosaic perfusion, focal hyperemia in a subset of pulmonary opacities, and focal oligemia associated with a subset of peripheral opacities [7]. We decided to study the incidence of some of these perfusion abnormalities in non-hospitalized patients diagnosed with mild COVID-19 infection.

**Fig. 2:**
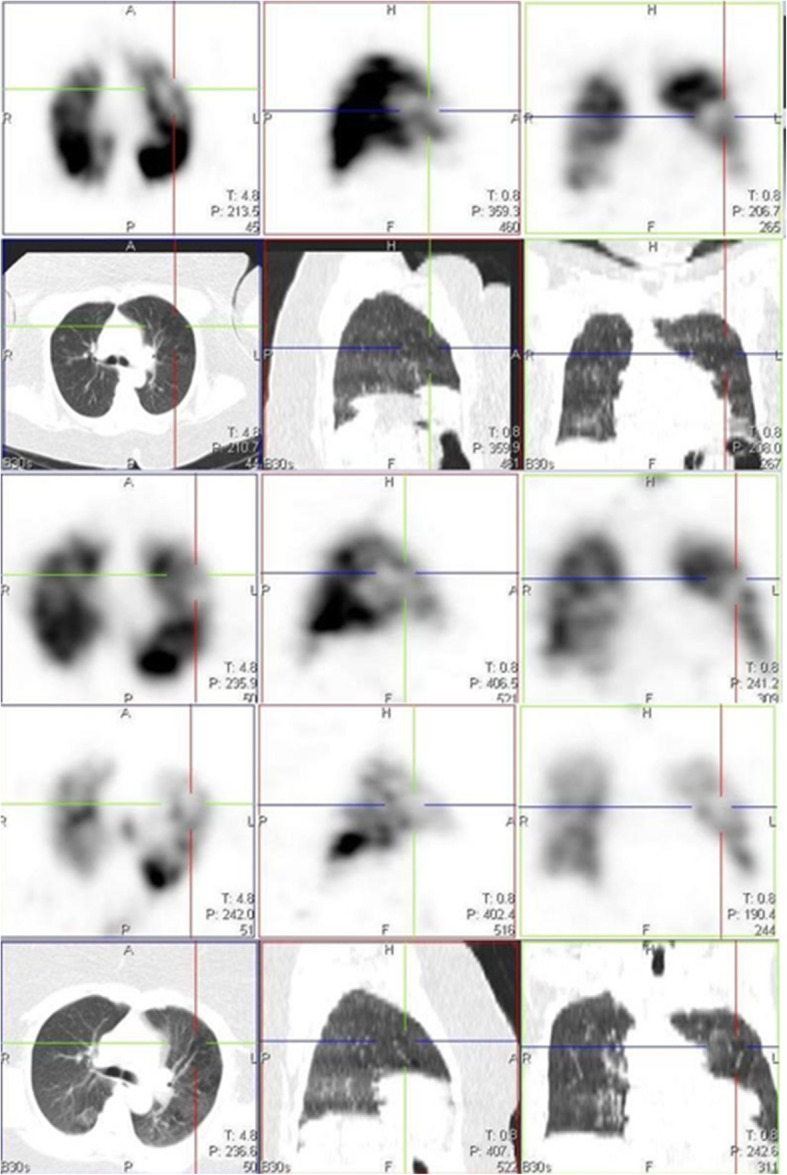
Images in rows 1 and 2 are perfusion only SPECT/CT images of a 46-year-old female, 14 days post COVID-19 infection, showing a perfusion defect in the left lung (crosshair), with associated mosaic hypoattenuation on CT seen in the first two rows. Images in rows 3, 4, and 5 are VQ SPECT/CT images acquired 3 months later, confirming matching defects with little or no reduction in defect size and persistent mosaic hypoattenuation on CT

A few studies have already reported the use of conventional CT or dual energy CT (DECT) pulmonary angiography in studying some of these perfusion abnormalities [2, 5–8]. Patelli et al. [2], in their study, demonstrated hypo perfusion in 3 patients with dyspnea, more than 1 month after the diagnosis of COVID-19 infection. Pulmonary thromboembolism was excluded in these patients and a suggestion of persistent microvascular defect was thought to be the reason for the hypo perfusion. Lang et al. [5], in their findings, also demonstrated mosaic perfusion pattern in 3 patients without PE. They proposed that there might be possible perfusion shunting due to increased perfusion to regions of inflammation and decreased perfusion to the area of normal lung parenchyma. Another study by Ling et al. [7] demonstrated hypo perfusion in areas with mosaic attenuation in 96% of their study population who had DECT. In our study, we had 17 cases with one or more perfusion defects not due to PE, but associated with mosaic attenuation on the CT images (Fig. 2).

**Fig. 3:**
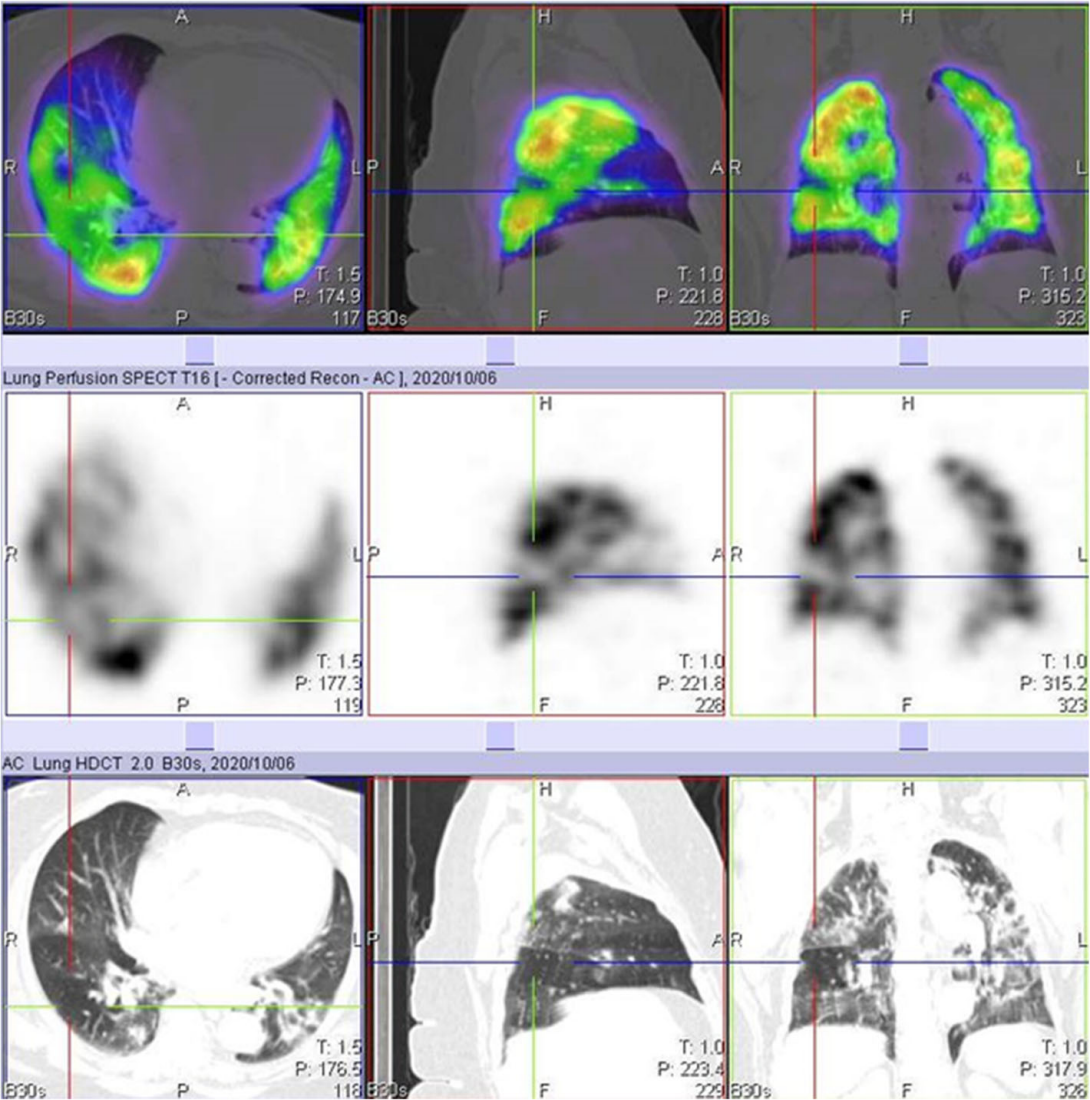
Perfusion only SPECT/CT images of a 42-year-old female, 13 days post COVID-19 infection, showing increased perfusion to areas of inflammation in the right lung, with a perfusion defect in an area without inflammation (crosshair) and associated mosaic hypoattenuation on CT

**Fig. 4:**
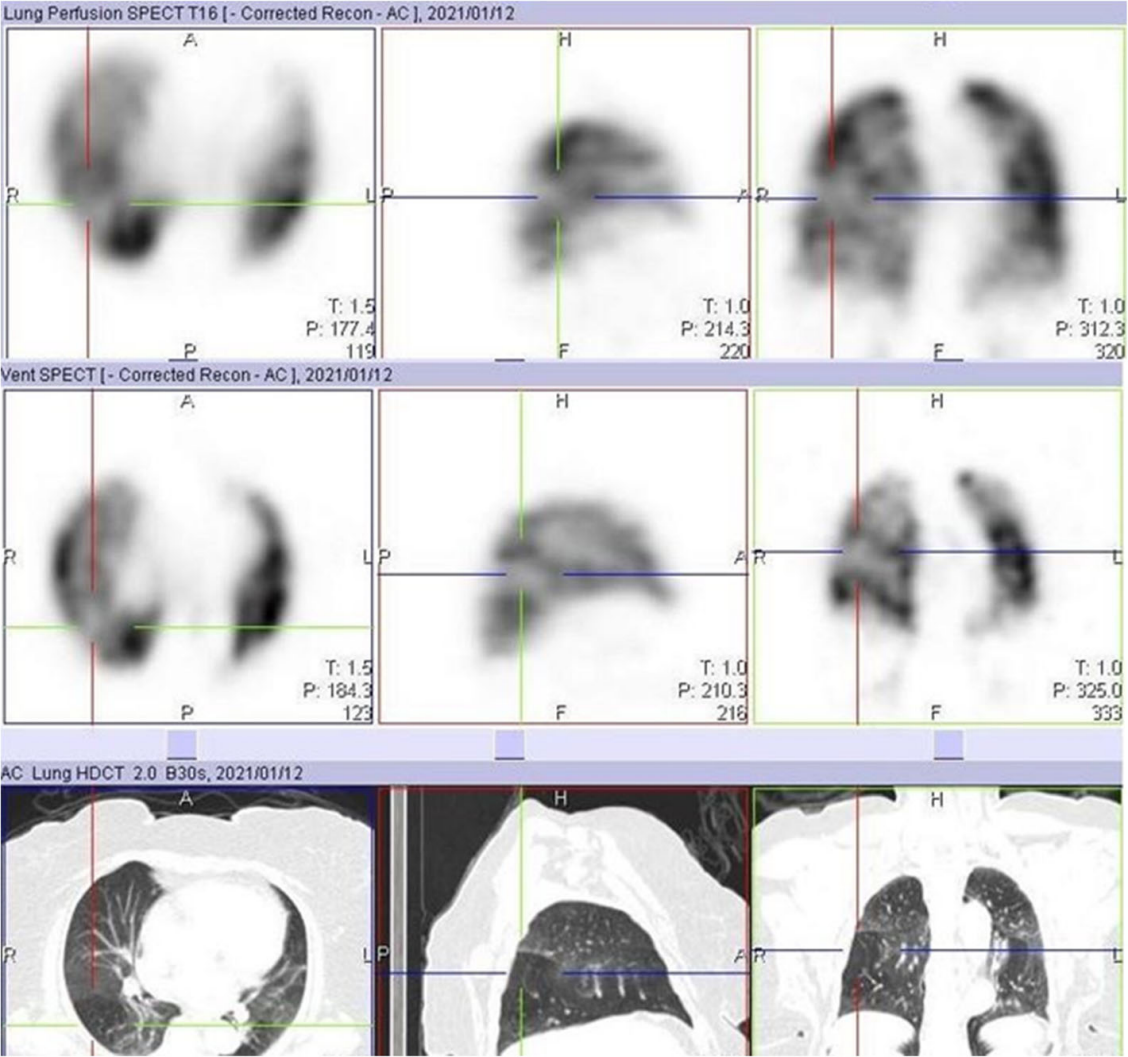
Ventilation perfusion SPECT/CT study of the same patient in Fig. 3, performed 3 months after her initial perfusion only SPECT/CT study. The previously seen defect on the initial perfusion only study in the right lung (crosshair) is matched

**Fig. 5:**
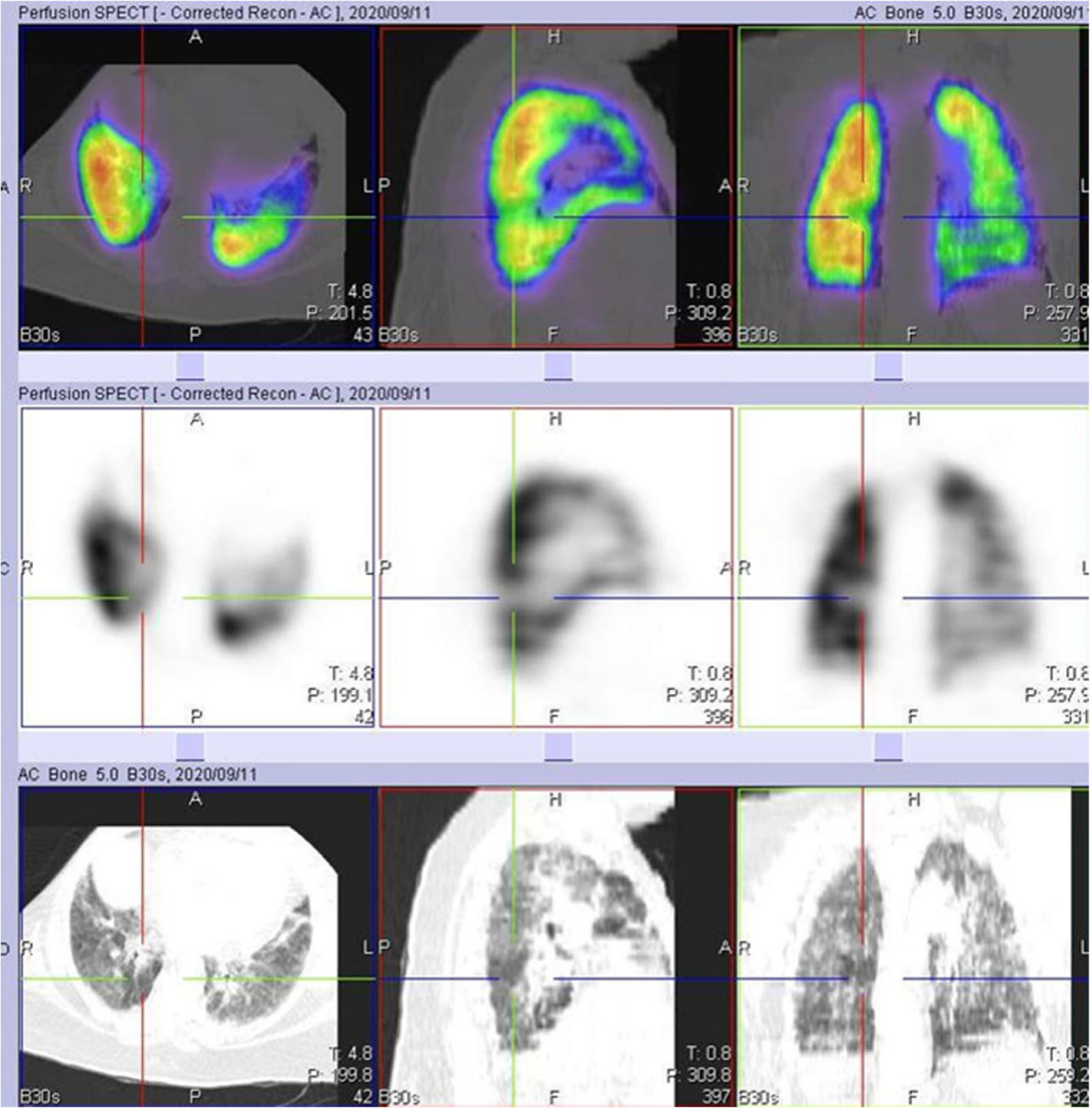
Perfusion only SPECT/CT image of a 32-year-old female, performed 18 days after testing positive for COVID-19 infection, showing a perfusion defect in the right lung (crosshair) that appears to be associated with shunting

**Fig. 6:**
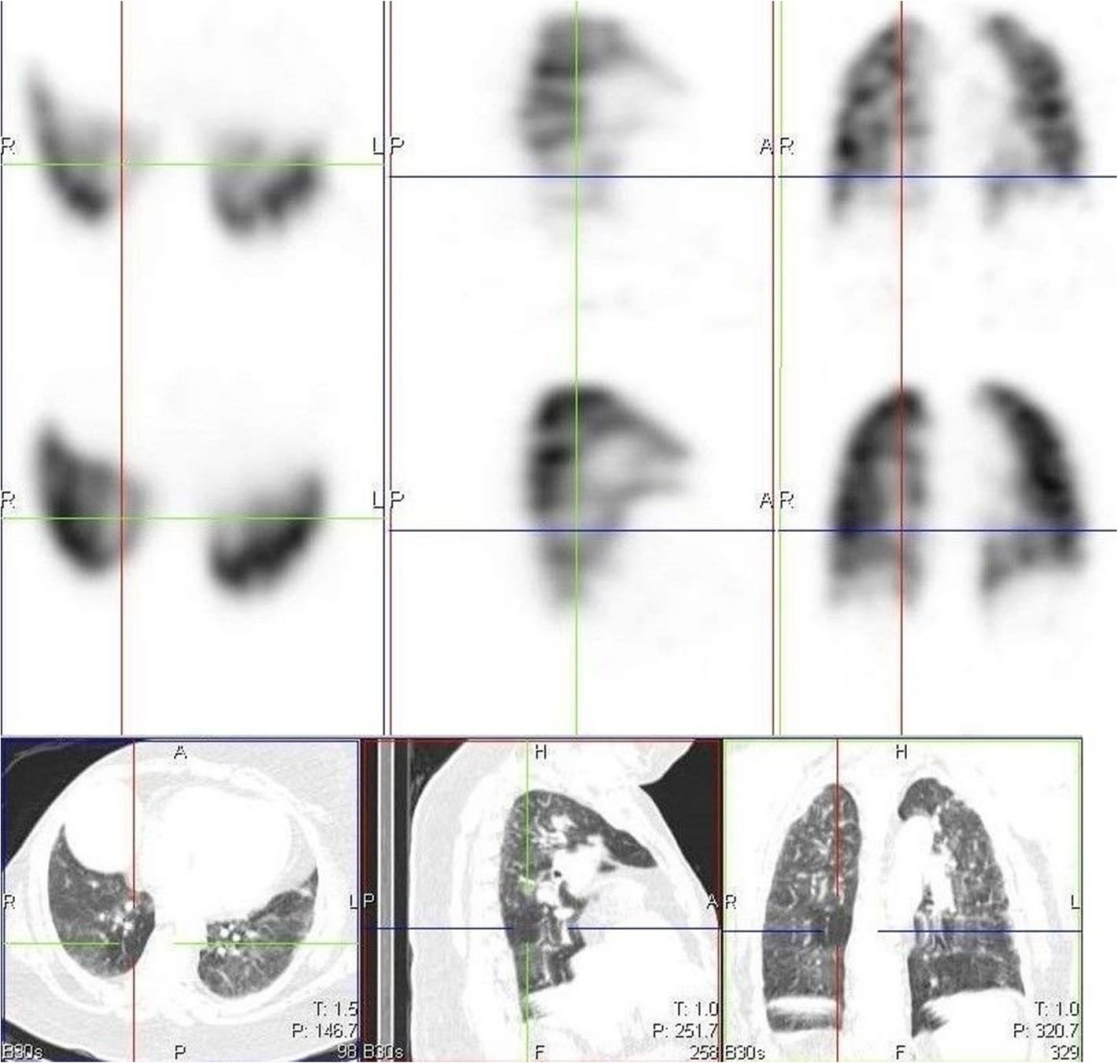
Ventilation perfusion SPECT/CT images performed over a year earlier before the COVID 19 pandemic in the same case in Fig. 5, showing matching of the defect and corresponding mosaic attenuation

We also identified 10 cases of increased perfusion to areas with associated inflammation and resulting perfusion defects, in areas with normal lung parenchymal (Fig. 3). This pattern is thought to have resulted from a loss of normal physiologic hypoxic vasoconstriction in areas with inflammation, leading to vasodilatation in this area and possible shunting of blood from areas without inflammation [5–8]. These studies also identified a possible VQ mismatch in these areas of hypoperfusion as a reason for some of the respiratory symptoms in COVID-19 patients. However, our study shows that these defects are matched on the ventilation study (Fig. 4). This pattern may not be specific to COVID-19 patients, as an earlier VQ SPECT/CT study performed in one of the patients in January 2019 showed a similar pattern (Figs. 5 and 6).

As earlier noted, 10 of the 17 cases with perfusion defects and mosaic attenuation not due to PE had associated increased perfusion to areas of inflammation. This most likely leads to possible shunting of blood from areas of the lung without inflammation. The probable cause of the perfusion defects and mosaic attenuation in the remaining 7 cases is uncertain. We hypothesize that this could either be due to microvascular thrombosis, small airway disease, persistent defect after resolution of a previous shunt, or a rare case of PE with a matching ventilation study.

**Fig. 7:**
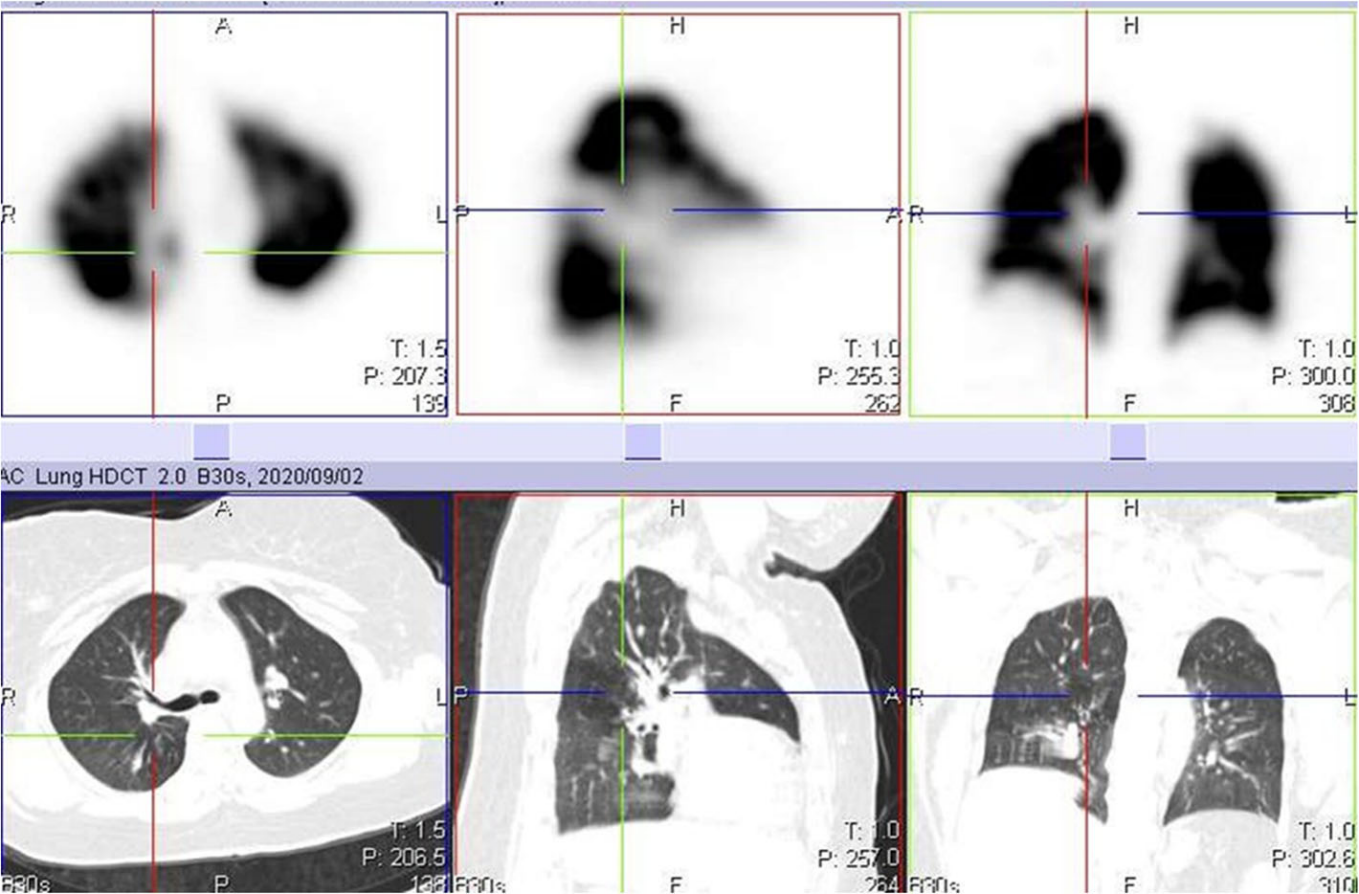
Perfusion only SPECT/CT study of a 40-year-old female, performed 12 days after testing positive for COVID-19 infection, showing a large perfusion defect in the right lung (crosshair). This was initially attributable to pulmonary embolism

**Fig. 8:**
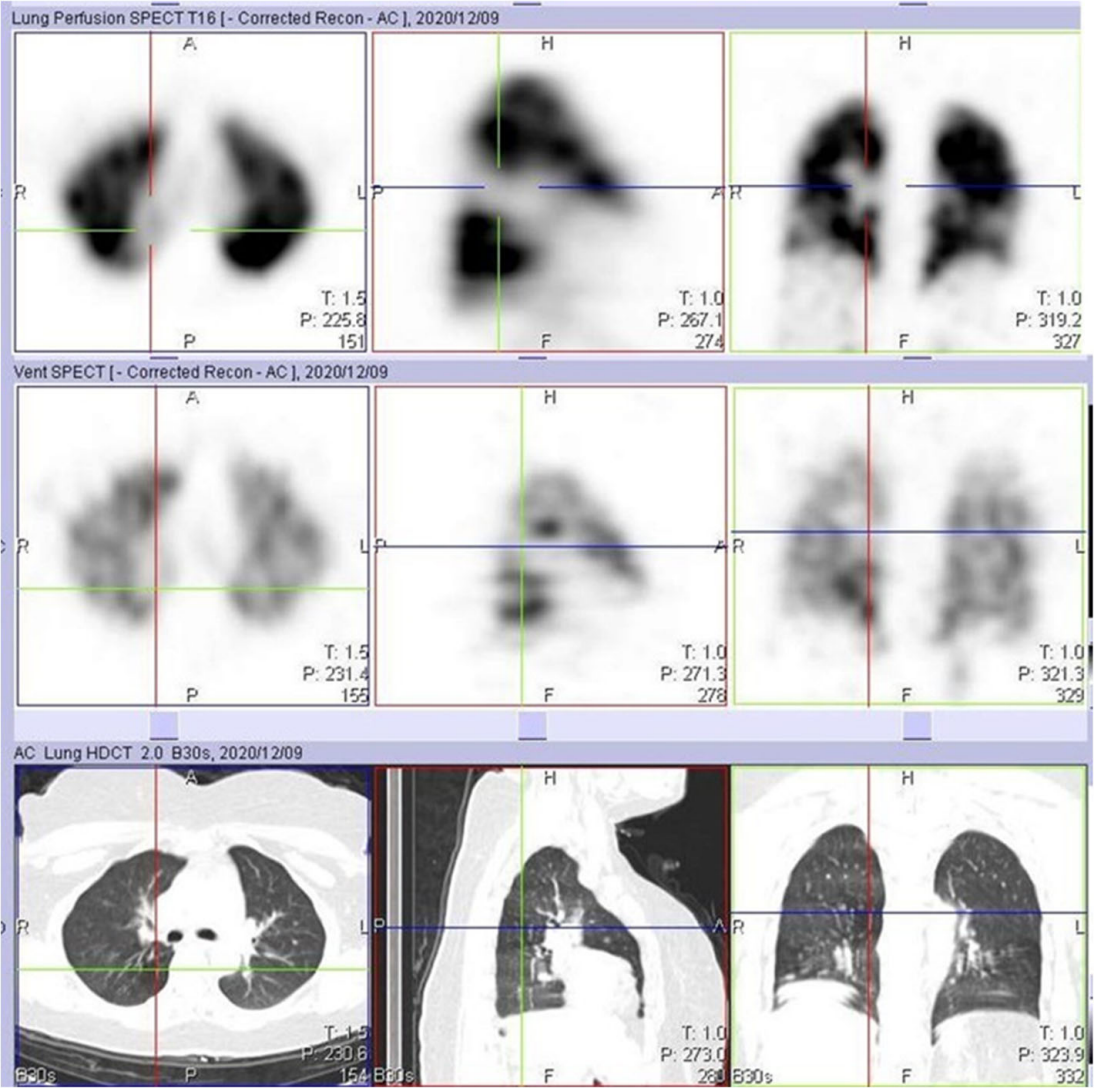
Ventilation perfusion SPECT/CT study of the same case in Fig. 7 performed 3 months after initiating anticoagulation showing matching, with no reduction in the size of the perfusion defect. Note the two unmatched perfusion defects in both lower lung zones

During the early parts of the COVID-19 pandemic, we omitted the ventilation part of the study, thereby minimizing the possibility of the spread of infection. However, we erroneously attributed some of these perfusion defects to PE, as the corresponding CT images did not show obvious parenchymal CT changes. By the time some of our patients had their follow-up study at 3 months, we had resumed the ventilation aspect of the study. These follow-up VQ studies performed 3 months after the initial perfusion only study confirmed that some of the earlier perfusion defects were matched (Figs. 7 and 8). This highlights the importance of the ventilation part of the study in patients with COVID-19 infection.

**Fig. 9:**
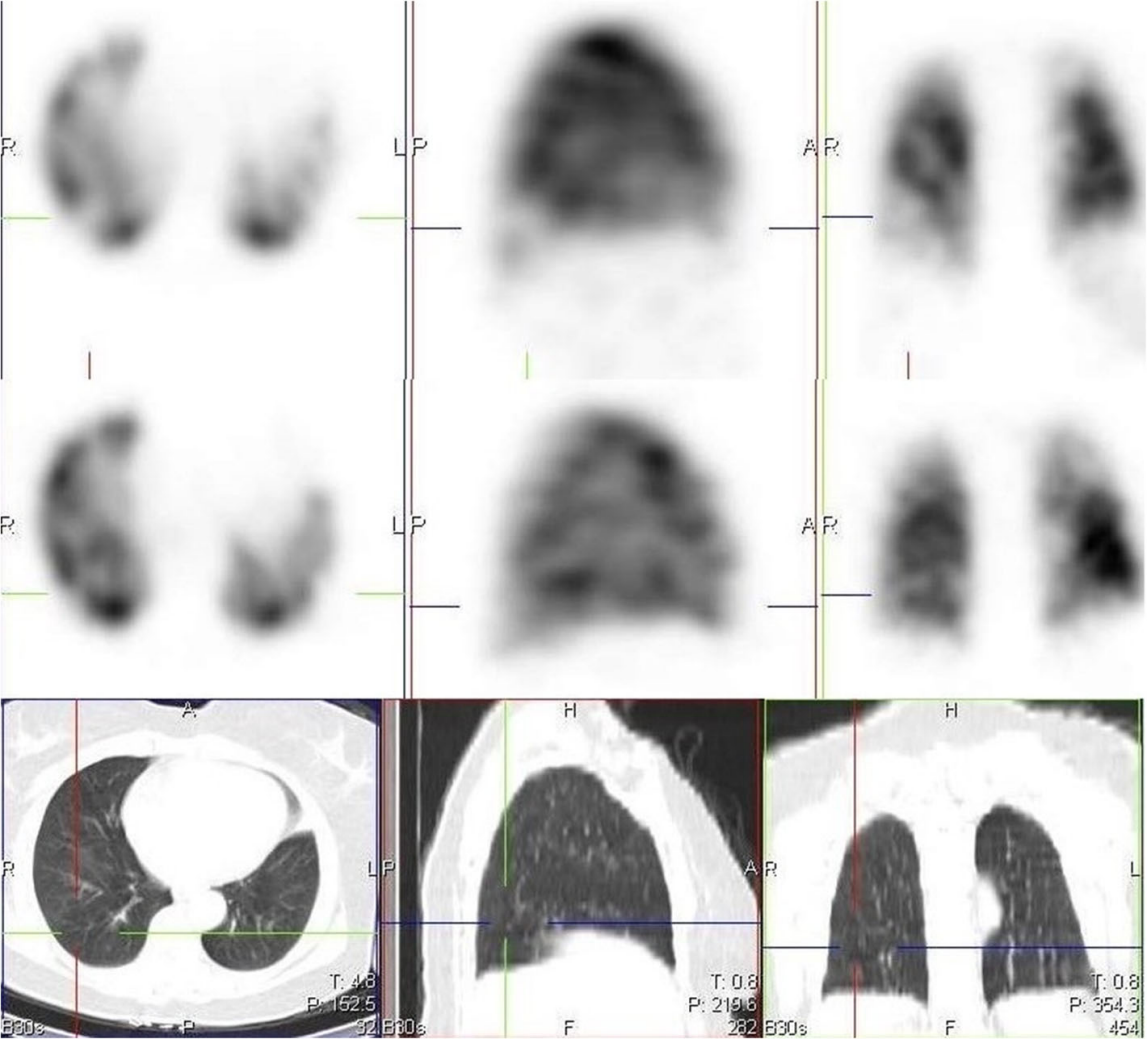
Ventilation perfusion SPECT/CT study showing an unmatched perfusion defect in the right lower lobe (crosshair). Note no corresponding mosaic hypoattenuation on CT

**Fig. 10:**
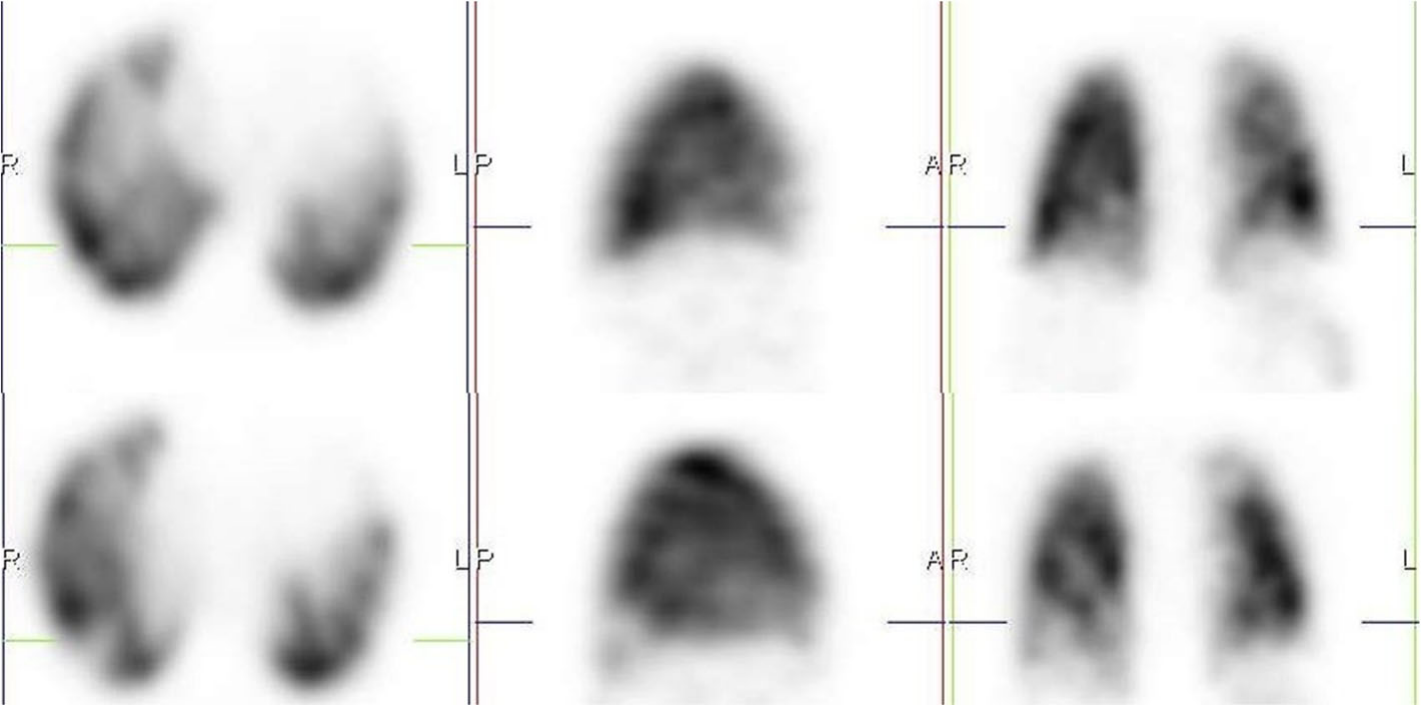
The perfusion SPECT image on top of the same case in Fig. 8 shows complete defect resolution in the right lower lobe when compared to the initial study

Pulmonary embolism is a known thrombotic complication that has been associated with COVID-19 infection [14]. Numerous studies have shown the incidence of PE in this condition [4, 14–17]. However, the category of most of the patients studied has been hospitalized patients with severe disease, with some of them being admitted into intensive care units [4, 14–20]. Our study population was that of non-hospitalized patients diagnosed with mild disease. There has not been enough data yet looking at the prevalence or incidence of PE in this group. There are, however, some case reports that have reported the occurrence of PE in non-hospitalized patients diagnosed with COVID-19 infection [21–24]. In our study, there were 20 (35.7%) cases with defects in keeping with PE (Fig. 9). Follow-up studies performed 3 months after initiation of anticoagulation in some of these patients demonstrated defect resolution (Fig. 10). We suspect that the significant number of cases with PE in our cohort was due to some of the peculiar characteristics of our study population. Our study population only included those patients with persistent or new onset respiratory symptoms and raised D-dimer levels. If we were to include all cases with mild disease, and without respiratory symptoms or raised D-dimer levels, we would most likely have a lower percentage with PE.

Of particular interest, we had a patient who had been symptomatic for about 90 days post de-isolation. The VQ SPECT/CT study performed revealed mismatched defects in keeping with PE. This goes to show that it is very possible that some of these patients might not be diagnosed at all or early enough with PE. This puts them at an increased risk of morbidity or mortality from a second embolus, pulmonary hypertension, or right ventricular failure.

It is known that there is a tendency to have a false-positive diagnosis of PE, with perfusion only SPECT/CT imaging [9, 25]. In our study, we had 2 cases that were wrongly diagnosed with PE. Their repeat VQ SPECT/CT study performed 3 months after initiating anticoagulation showed matching defects, with no sign of defect resolution. Again, this highlights the importance of the ventilation part of the study, as it improves the specificity of a perfusion only SPECT/CT study.

**Fig. 11:**
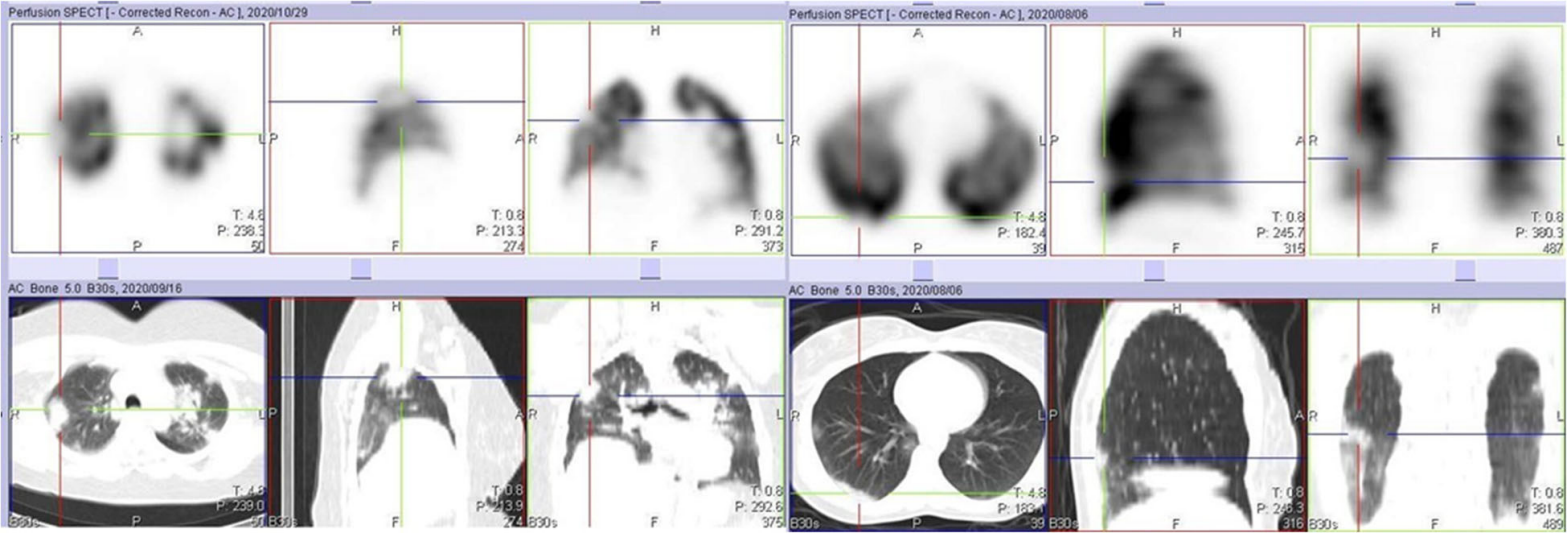
Perfusion only SPECT/CT images of two cases showing perfusion defects in areas with COVID pneumonia

In our study, 42.9% of the population had associated COVID pneumonia on their CT images. This has been well described in the literature using various reporting patterns [2, 7]. As described earlier, we demonstrated how some of these inflammatory changes in the lungs were associated with increased perfusion. However, in this study, we also observed that 6 (10.7%) of the cases had perfusion defects in areas associated with COVID pneumonia changes (Fig. 11).

We had 2 cases that presented at 3 months after de-isolation, with a false-negative CT pulmonary angiography (CTPA) study. The VQ studies performed on the same day as the CTPA study revealed mismatched perfusion defects in keeping with PE. These 2 cases in particular had persistent respiratory symptoms for over 3 months after de isolation, making chronic PE more likely. The sensitivity of CTPA in the diagnosis of chronic PE is low [13]. This could be the reason why these cases were missed in the CTPA study. It also highlights the potential advantage a VQ study has over a CTPA study performed in this cohort of patients, as a number of them might be investigated in the chronic phase.

Patients with severe COVID-19 infection are likely to be admitted to hospitals, including intensive care units, and placed on prophylactic anticoagulation. They also have a higher chance of being investigated for PE. On the other hand, patients with mild disease are unlikely to be hospitalized and readily investigated for PE. This increases the chances of a missed or delayed diagnosis of PE in this category of patients. We, therefore, recommend that, irrespective of the disease severity or need for hospitalization, all patients with COVID-19 infection, raised D-dimer levels, and persistent or new onset respiratory symptoms need to be further investigated for perfusion abnormalities such as PE. Although the clinical significance of the other causes of perfusion abnormalities in these patients might not be clear, there is a chance that long-term complications might occur if untreated.

We believe that VQ SPECT/CT study is a very good modality in the identification and follow-up of these perfusion abnormalities. The CT portion of the study is also useful in identifying those patients with mosaic attenuation or associated COVID-19 pneumonia. These findings may likely determine the specific management plan for each of these patients.

Some limitations were noted. With a retrospective study design, we could not rule out the existence of some of these lung perfusion abnormalities in our patient population before their COVID-19 infection. We did not identify other possible risk factors for perfusion abnormalities in these patients. We, therefore, recommend that proper prospective studies be carried out in these patients, as this will show the true incidence of perfusion abnormalities in patients with mild COVID 19 infection.

## Conclusion

Lung perfusion abnormalities are not an uncommon occurrence in non-hospitalized patients diagnosed with mild COVID-19 infection. Ventilation perfusion SPECT/CT imaging shows promise in identifying the exact type of perfusion abnormalities and lung parenchymal changes present in a sub set of these patients, with raised D-dimer levels and persistent or new onset respiratory symptoms. Although we still do not know the clinical relevance of some of these perfusion abnormalities, VQ SPECT/CT imaging may be a good tool in guiding further management and monitoring response to treatment.

## Data Availability

All data and material of the article are readily available.

## Abbreviations

SARS-CoV-2: Severe acute respiratory syndrome coronavirus 2
COVID-19: Coronavirus disease 2019
ACE 2: Angiotensin-converting enzyme 2
PE: Pulmonary embolism
VQ: Ventilation perfusion
SPECT/CT: Single photon emission computed tomography/computed tomography
HSREC: Health Sciences Research Ethics Committee
^99m^Tc-DTPA: Technetium 99 metastable diethylenetriamine pentacetate
MAA: Macro-aggregated albumin
LEHR: Low energy high resolution
OR: Odds ratios
CI: Confidence interval
IQR: Interquartile range
DECT: Dual energy computed tomography
CTPA: Computed tomography pulmonary angiography
